# Serum Fibroblast Growth Factor 21 Levels Are Correlated with the Severity of Diabetic Retinopathy

**DOI:** 10.1155/2014/929756

**Published:** 2014-04-15

**Authors:** Yuan Lin, Ye-cheng Xiao, Hong Zhu, Qing-yan Xu, Lei Qi, Yu-bin Wang, Xiu-juan Li, Ma-li Zheng, Rui-sheng Zhong, Yi Zhang, Xiang-dong Xu, Bo-le Wu, Zhu-mei Xu, Xiang-hong Lu

**Affiliations:** ^1^Department of Ophthalmology, Xiamen Hospital of Traditional Chinese Medicine, Xiamen 361000, China; ^2^Department of Ophthalmology, The Third Affiliated Hospital, Fujian Traditional Chinese Medicine University, Xiamen 361000, China; ^3^Key Laboratory of Biotechnology Pharmaceutical Engineering, School of Pharmacy, Wenzhou Medical University, Wenzhou 325035, China; ^4^Department of Endocrinology, The First Affiliated Hospital, Wenzhou Medical University, Wenzhou 323000, China; ^5^Department of Ophthalmology, Lishui People's Hospital, Wenzhou Medical University, Lishui 323000, China

## Abstract

The aim of the study was to assess serum fibroblast growth factor 21 (FGF21) concentrations in Chinese type 2 diabetic patients with and without retinopathy and to assess the association between FGF21 and the severity of retinopathy. 117 diabetic patients were compared with 68 healthy controls. Fasting blood glucose, serum total cholesterol, serum triglycerides, serum insulin, and serum FGF21 levels were estimated. FGF21 concentrations in the patients were significantly higher than those in control. In the patient group there was a significant positive correlation between FGF21, insulin level, and homeostasis model assessment index. Serum FGF21 concentrations in patients with proliferative diabetic retinopathy or nonproliferative diabetic retinopathy were significantly higher than those in patients without diabetic retinopathy. When the presence of diabetes was defined as the final variable in the conditional logistic regression model with the FGF21 concentration as the continuous variable, FGF21 was significantly involved in the model. This study shows that the increase in serum concentration of FGF21 was associated with the severity of diabetic retinopathy and suggests that FGF21 may play a role in the pathogenesis of diabetic retinopathy and its degree.

## 1. Introduction

Diabetes mellitus continues to be a tremendous burden throughout the world and is a significant cause of morbidity and mortality. Diabetic retinopathy is a microvascular complication of diabetes correlated with chronic hyperglycemia [1]. Diabetic retinal neovascularization is considered to be a major consequence of retinal ischemia caused by capillary occlusion, and the mechanism of its development is not clear [2, 3]. Diabetic retinopathy progresses from mild nonproliferative abnormalities (characterized by increased vascular permeability) to moderate and severe nonproliferative diabetic retinopathy (NPDR) (characterized by vascular closure) and then to proliferative diabetic retinopathy (PDR) (characterized by the growth of new blood vessels on the retina and posterior surface of the vitreous) [4, 5]. Macular edema (characterized by retinal thickening from leaky blood vessels) can be developed at any stage of retinopathy. The new blood vessels of PDR and contraction of all accompanying fibrous tissues can distort the retina and lead to fractional retinal detachment, producing severe and irreversible vision loss [6].

Despite improvements in glycemic control, the incidence of blindness in diabetic patients has progressively increased. Pharmacological and nonpharmacological interventions that target growth factor pathways are currently being investigated as a potential approach for the prevention and treatment of diabetic retinopathy [7, 8]. Many growth factors are implicated in the development of vascular pathologies in diabetic retinopathy, including vascular endothelial growth factor (VEGF), platelet derived growth factor (PDGF), insulin-like growth factor (IGF), and fibroblast growth factor (FGF) [9–12].

The role of these growth factors in the pathogenesis of diabetic retinopathy is complex. The fibroblast growth factor family is composed of 22 members with a wide range of biological functions, including cell growth, development, angiogenesis, and wound healing [13]. FGF21 is a member of the endocrine FGF subfamily, which is expressed predominantly in the liver and stimulates glucose uptake through the induction of GLUT1 in adipocytes [14].* In vivo* treatment with FGF21 results in the amelioration of glucose and regulates lipid metabolism in both murine and nonhuman primate models of diabetes and obesity [15]. Taken together, these findings demonstrate that FGF21 plays an important role in the regulation of glucose and lipid metabolism and also suggest that FGF21 exhibits the therapeutic characteristic necessary for the effective treatment of diabetes and obesity.

Recent human studies indicated that increased serum levels of FGF21 are found in obese individuals and subjects with metabolic syndrome and type 2 diabetes mellitus [16–18]. Whether FGF21 is closely associated with the pathology of retinopathy in diabetic patients remains unclear. To explore the physiological and pathological relevance of FGF21 in patients with diabetic retinopathy, we measured the serum concentrations of FGF21 in Chinese subjects and analyzed its association with a cluster of metabolic parameters that related to the changes seen in retinopathy.

## 2. Methods

### 2.1. Study Subjects

The study population consisted of 117 outpatients with diabetic retinopathy and 68 healthy individuals. The 117 patients with diabetic retinopathy were recruited from outpatient ophthalmology clinics at Xiamen Hospital of Traditional Chinese Medicine, Xiamen. Patients recruited into this study were aged ≥25 between October 8, 2009, and May 11, 2012. Patients who were seen in the retina, glaucoma, cornea, and comprehensive ophthalmology clinics during the enrollment period were considered potential study subjects. After undergoing routine ophthalmic examination, subjects were recruited and divided into the four groups, based on their diabetes status and retinopathy findings. The control group is the no diabetic retinopathy (no DR) group consisting of subjects with type 2 diabetes but no evidence of diabetic retinopathy, such as microaneurysms, cotton-wool spots, intraretinal hemorrhages, or macular edema. The nonproliferative diabetic retinopathy (NPDR) group comprised patients with evidence of retinopathy such as microaneurysms, cotton-wool spots, intraretinal hemorrhages, or macular edema but no evidence of retinal or iris neovascularization. Lastly, the proliferative diabetic retinopathy (PDR) group consisted of subjects with neovascularization on the optic disc, retina, or iris, with or without vitreous hemorrhage or prior panretinal photocoagulation. When the diabetic retinopathy was asymmetric, the subject was assigned to the group according to the eye with the worst retinopathy. Subjects were excluded if they had type 1 diabetes, were younger than 18, or were older than 90 years of age.

Sixty-eight healthy volunteers served as the control group. They were matched with the diabetic patients according to age, BMI, blood pressure, and sex. They underwent routine physical and laboratory evaluations to ensure that none had diabetes or other metabolic, hepatic, or renal diseases. In addition, none of the healthy volunteers had a family history of hypertension or diabetes. Subjects in this group were not excluded if they had other forms of ocular disease, such as uveitis or macular degeneration. All individuals gave informed consent to participate in the study, which was approved by the Ethics Committee of Fujian Traditional Chinese Medicine University.

### 2.2. Biochemical Measurements

Once enrolled in the study, subjects completed a medical history questionnaire and had their blood drawn. Patients were not required to fast prior to this blood draw. One blood sample was immediately tested for Glycated hemoglobin (HbA1c). Other whole blood, serum, and plasma samples were frozen at −80°C. Serum concentrations of glucose (glucose oxidase peroxidase calorimetric method) and creatinine (Jaffe' method) were determined using a Technicon Dax-48 system analyzer (Miles, Tarrytown, NY, USA). HbA1c was determined by inhibition of latex agglutination, using a DCA 2000 analyzer (Bayer, Elkhart, IN, USA). Microalbuminuria was detected by an immunoturbidimetric method with an Urinpak Micro Albumin immunokit (Miles, Tarrytown, NY, USA). Total cholesterol, high-density lipoprotein cholesterol, and triglyceride levels were determined by an enzymatic colorimetric method with an Olympus AU 600 autoanalyzer, using reagents from Olympus Diagnostics GmbH (Hamburg, Germany). Low-density lipoprotein (LDL) cholesterol level was calculated by Friedewald's formula.

The basal serum concentration of insulin was determined by the coated-tube method (Diagnostic Products Corporation, Los Angeles, CA, USA). In brief, a homeostasis model of assessment insulin resistance score (HOMA-IR) was computed with the following formula: (HOMA-IR) = fasting plasma glucose (mg/dL) × immunoreactive insulin (*μ*U/mL)/405 [19]. Serum levels of FGF-21 (Biovendor, Modrice, Czech Republic) and C-reactive protein (CRP, R&D, USA) were determined with commercially available enzyme-linked immunosorbent assays according to the manufacturers' instructions.

### 2.3. Statistical Analysis

Data were processed using the SPSS 11.5 statistical package. Results are reported as mean ± SD. Levene's test was used to evaluate the distribution characteristics of the variables. Differences between diabetic and control groups were tested for significance by *t*-tests, Mann-Whitney *U* tests and *χ* ^2^-tests. The relationship between variables was analyzed by Pearson's correlation. The results were also analyzed by one-way analysis of variance, Bonferroni-adjusted Mann-Whitney *U* tests, and *t*-tests for the comparison of subgroups. We used the odds ratio (OR) as a measure of association between outcome and exposure and estimated the adjusted odds ratios in conditional logistic regression analyses.

Receiver operating characteristics (ROC) curve analysis was performed to determine a threshold concentration of FGF21 for the development of diabetic retinopathy. *P* < 0.05 was considered statistically significant.

## 3. Results

### 3.1. Subject Characteristics

The clinical and laboratory data of all participants are shown in Table 1. No significant differences in age and systolic and diastolic blood pressures were observed between the patient and control group. The mean body mass index (BMI) was statistically lower in the control group than the patient groups. The insulin, fasting blood glucose, and HOMA-IR indexes of the patients were significantly greater, while the FGF21 concentrations were also higher than those of the controls (*P* < 0.001 for all). The percentage of subjects with macrovascular disease was significantly lower in the no DM group, compared to the no DR, NPDR, and PDR groups.

There was a significant positive correlation between serum FGF21 and insulin or HOMA-IR index in the group of patients (*r* = 0.498, *P* < 0.001, and *r* = 0.341, *P* < 0.05, resp.). In addition, serum FGF-21 levels correlated with fasting glucose, fasting insulin, and HOMA-IR after adjustment for BMI, but no significant correlation was found between serum FGF-21 levels and lipid parameters, HOMA-IS. Furthermore, serum FGF-21 levels correlated with macrovascular disease, but the significant differences between the FGF21 concentrations of the subgroups were not affected by the sex distribution.

### 3.2. FGF-21 Analysis

**Figure 1:**
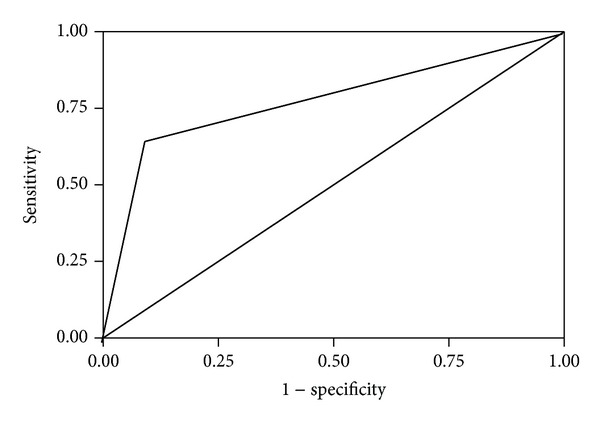
ROC curve of serum FGF21 concentrations for occurrence of diabetic retinopathy. The estimated cut-off value of FGF21 is 550 pg/mL, with 86.5% sensitivity and 75% specificity for the existence of diabetic retinopathy (area under the curve = 0.776, *P* > 0.05). Diagonal segments were produced by ties.

After the patients were subdivided according to severity of retinopathy, age, sex, and concentrations of fasting blood glucose, HbA1c, and insulin, the HOMA-IR indexes were similar for each subgroup (Table 2). Serum FGF21 concentrations in patients with PDR (*n* = 49; 669.4 ± 89.2 pg/mL) or NPDR (*n* = 34; 631.9 ± 73.8 pg/mL) were significantly higher than those in patients without retinopathy (*n* = 34; 326.8 ± 81.6, *P* < 0.001). Serum FGF21 concentrations in the patients with PDR were not significantly higher than those in patients with NPDR (*P* = 0.671; Table 2). There was a significant positive correlation between serum FGF-21 levels and macrovascular disease in no DR, NPDR, and PDR patients. The effects of serum FGF21 concentrations on diabetes and diabetic retinopathy were assessed by conditional logistic regression analysis, after adjustment for the other parameters (HOMA-IR, insulin, HbA1c, FGF21, and glucose) by matching. When the presence of diabetes was defined as the final variable in the conditional logistic regression model with the FGF21 concentration as the continuous variable, FGF21 was significantly involved in the model (*P* = 0.001; Table 3). ROC curve analysis was performed in order to establish a threshold FGF21 concentration for the existence of diabetic retinopathy. However, there was not a statistically significant cut-off value of serum FGF21 for the condition (Figure 1).

## 4. Discussion

Diabetic retinopathy is the major cause of irreversible blindness in patients of working age throughout the world [20]. Diabetes reduces angiogenic potential in the retinal microvasculature, suggesting that, as well as inactive proliferative retinopathy, the angiogenic potential of retinal microvascular endothelial cells is significantly compromised by the diabetic state [21]. In China, through early detection of the condition through screening programs, patients who receive treatment are less likely to become blind from proliferative changes and maculopathy. However, a screening program is effective only if patients at risk can be identified and persuaded to attend. Therefore, a reliable, easily performable, and inexpensive screening method is urgently required for the early detection of diabetic retinopathy [22]. The association of FGF21 with the severity of diabetic retinopathy may open up future projects on the early detection and prevention of the condition.

FGF21 is a unique member of the FGF family and plays a significant role in regulating glucose and lipid metabolism, including stimulating glucose uptake insulin-independently, improving hyperglycemia and dyslipidemia [15]. This study shows that serum FGF21 concentrations are higher in diabetic patients compared to controls and are higher in diabetic patients with DR than those without DR, suggesting that serum FGF21 may play a role in the pathogenesis of DR. In the present study, our data also indicate that serum FGF21 levels are significantly associated with insulin, HOMA-IR index, or macrovascular disease in NPDR and PDR subjects, suggesting that elevated FGF21 levels are closely related to the injury of capillary and the change of retinal function in diabetic retinopathy patients. However, the mechanisms responsible for the elevation of FGF21 concentration with the progression of diabetic retinopathy are not fully understood. FGF21 is expressed predominantly in liver and adipose tissue [23] and plays an important role in regulating lipid and energy metabolism [24, 25]. We speculate that the paradoxical increase in diabetic retinopathy patients is a compensatory mechanism to counteract metabolic stress. FGF21 resistance had been found in obesity and in cardiovascular failure, leading to compensatory upregulation of adiponectin [26, 27]. Based on these findings, we propose that the mechanism of increased FGF21 levels in diabetic retinopathy is similar to those observed in hyperglycemia-associated resistance to adiponectin. In response to endothelial dysfunction, serum adiponectin or FGF21, both as beneficial hormones to diabetes, may be compensatorily increased to repair microvascular legions involved in retinopathy.

The major finding in the present study was that serum concentrations of FGF21 were increased in diabetic patients but nonproportional to the severity of retinopathy. Although many factors are reported to affect the progression of diabetic retinopathy [28–30], there is general agreement that the duration of diabetes and the severity of hyperglycemia are the major risk factors [8]. Thus, intensive treatment only results in delaying the procession of diabetic retinopathy and cannot prevent its development completely [31]. From the findings of the Diabetes Control and Complications Trial [22], susceptibility to diabetic retinopathy is influenced by genetic factors, in addition to other well-known causes of the condition. In recruiting the patients for this study, we were careful to ensure that all subgroups were similar, in terms of their body weight and other factors known to contribute to the development of retinopathy, such as HbA1c, time elapsed since diagnosis of diabetes, and coexistence of other significant health problems. The positive relationship between FGF21 concentration and retinopathy observed in this study was not accompanied by any obvious difference between the retinopathy subgroups with respect to factors promoting microangiopathy. According to the results of conditional logistic regression analysis, FGF21 is likely to be one of the major contributors to the pathogenesis of both type 2 diabetes and diabetic retinopathy. The results of the ROC curve analysis did not provide a threshold concentration of FGF21 for the existence of diabetic retinopathy and thus a predictive value cannot be assigned to FGF21 concentrations in relation to the development of the condition. This supposition therefore requires investigation in much larger sample groups.

However, there is no doubt that the limitations of FGF21 in diabetic retinopathy still need further study and investigation. The number of subjects in the study population was barely adequate to permit clear estimations to be made about the association of serum FGF21 concentrations with the severity of the diabetic retinopathy. It is like that with larger numbers of patients it would be possible to obtain a threshold for FGF21 concentration that is significant for the development of diabetic retinopathy. In addition, as a case-control design was used for the study used, it is not easy to accurately predict whether the high FGF21 concentrations preceded the retinopathy or vice versa. Future cohort studies will help provide more information on this.

In summary, the results from the present study suggest that serum FGF21 concentrations are higher in patients with type 2 diabetes than in age-, BMI-, and sex-matched controls. These concentrations are higher in patients with diabetic retinopathy than in those without it and are involved in the generation of diabetes and diabetic retinopathy. Future prospective studies with greater numbers of patients are recommended to establish a direct relationship between serum FGF21 concentrations and the severity of vascular complications.

## Figures and Tables

**Table 1:** Clinical and laboratory features of the patient and control groups. Values are means (±SD) or number (%).

	Control (*n* = 68)	Patients (*n* = 117)	*P* value
Age (years)	58.5 ± 11.3	60.3 ± 10.2	0.343
Sex (M/F)	37/31	63/54	0.362
BMI (kg/m^2^)	25.7 ± 6.8	32.9 ± 7.9	<0.001
Systolic BP (mmHg)	129.88 ± 11.58	133.69 ± 8.32	0.462
Diastolic BP (mmHg)	83.63 ± 3.69	84.33 ± 4.39	0.653
Total cholesterol (mmol/L)	11.55 ± 2.13	11.39 ± 1.57	0.852
Triglycerides (mmol/L)	6.93 ± 1.74	7.21 ± 1.14	0.578
LDL cholesterol (mmol/L)	6.77 ± 3.61	6.54 ± 0.75	0.842
HDL cholesterol (mmol/L)	2.33 ± 0.58	2.31 ± 0.55	0.673
Insulin (*μ*U/mL)	6.55 ± 1.03	13.13 ± 8.74	<0.001
HOMA-IR	1.33 ± 0.34	3.11 ± 0.84	<0.001
HbA1c (%)	5.9 ± 0.7	8.0 ± 0.8	<0.001
FPG (mmol/L)	4.54 ± 1.53	7.66 ± 1.47	<0.001
Macrovascular disease	13 (13%)	39 (36%)	<0.001
FGF21* (pg/mL)	125.9 ± 39.3	542.3 ± 80.5	<0.001

DM: diabetes mellitus, DR: diabetic retinopathy, NPDR: nonproliferative diabetic retinopathy, PDR: proliferative diabetic retinopathy, BP: blood pressure, and FPG: fasting plasma glucose. Macrovascular disease represents coronary disease, cerebrovascular disease, and peripheral vascular disease.

*Bonferroni-adjusted *t*-test: *P* = 0.001 compare with Control.

**Table 2:** Clinical and laboratory features of type 2 diabetic patients with and without retinopathy. Values are means (±SD) or number (%).

	NO DR (*n* = 34)	NPDR (*n* = 34)	PDR (*n* = 49)	*P* value
Age (years)	59.4 ± 10.2	61.3 ± 10.1	59.2 ± 12.7	0.716
Sex (M/F)	20/14	22/12	21/28	0.964
Systolic BP (mmHg)	134.29 ± 12.41	129.77 ± 9.18	131.78 ± 6.70	0.268
Diastolic BP (mmHg)	85.77 ± 3.91	81.78 ± 2.47	83.68 ± 2.35	0.219
Total cholesterol (mmol/L)	11.55 ± 1.61	11.66 ± 1.23	10.79 ± 2.15	0.254
Triglycerides (mmol/L)	7.32 ± 1.32	7.49 ± 0.81	7.15 ± 1.56	0.288
LDL cholesterol (mmol/L)	6.63 ± 0.89	6.46 ± 0.79	6.31 ± 0.61	0.421
HDL cholesterol (mmol/L)	2.21 ± 0.44	2.31 ± 0.57	2.39 ± 0.77	0.216
BMI (kg/m^2^)	32.3 ± 9.1	32.7 ± 7.3	33.1 ± 5.9	0.684
Insulin (*μ*U/mL)	10.70 ± 3.13	12.08 ± 4.18	16.47 ± 4.59	0.076
HOMA-IR	2.96 ± 0.77	3.18 ± 0.60	3.25 ± 1.13	0.116
HbA1c (%)	7.7 ± 1.2	8.1 ± 0.3	8.3 ± 1.7	0.638
FPG (mmol/L)	7.52 ± 1.23	7.78 ± 1.38	7.68 ± 1.27	0.087
Macrovascular disease	6 (14%)	14 (33%)	19 (44%)	<0.001
FGF21* (pg/mL)	326.8 ± 81.6^∗#^	631.9 ± 73.8^†^	669.4 ± 89.2	<0.001

Bonferroni-adjusted *t*-test: *P = 0.01 compared with nonproliferative DR (NPDR), ^#^ *P* = 0.001 compared with proliferative DR (PDR), and ^†^ *P* = 0.671 compared with PDR.

**Table 3:** Odds ratios (OR) and 95% confidence intervals (CI) for associations of FGF21 with diabetes (*n* = 117) and retinopathy (*n* = 83).

	OR (95% CI)
Diabetes	0.71 (0.62 to 0.81)
Retinopathy	0.73 (0.64 to 0.85)
